# Did the universal zero-markup drug policy lower healthcare expenditures? Evidence from Changde, China

**DOI:** 10.1186/s12913-021-07211-8

**Published:** 2021-11-06

**Authors:** Zixuan Peng, Chaohong Zhan, Xiaomeng Ma, Honghui Yao, Xu Chen, Xinping Sha, Peter C. Coyte

**Affiliations:** 1 Institute of Health Policy, Management & Evaluation, Dalla Lana school of public health, University of Toronto, Toronto, Canada; 2 Xiangya Hospital, Central South University, Changsha, China; 3 Department of Learning, Informatics, Management and Ethics, Karolinska Institute, Stockholm, Sweden; 4 School of Social Science and Public Policy, King’s College of London, London, UK

**Keywords:** Health policy, Health care reform, Healthcare financing

## Abstract

**Background:**

The zero-markup drug policy (also known as the universal zero-markup drug policy (UZMDP)) was implemented in stages beginning with primary healthcare facilities in 2009 and eventually encompassing city public hospitals in 2016. This policy has been a central pillar of Chinese health reforms. While the literature has examined the impacts of this policy on healthcare utilization and expenditures, a more comprehensive and detailed assessment is warranted. The purpose of this paper is to explore the impacts of the UZMDP on inpatient and outpatient visits as well as on both aggregate healthcare expenditures and its various components (including drug, diagnosis, laboratory, and medical consumables expenditures).

**Methods:**

A pre-post design was applied to a dataset extracted from the Changde Municipal Human Resource and Social Security Bureau comprising discharge data on 27,246 inpatients and encounter data on 48,282 outpatients in Changde city, Hunan province, China. The pre-UZMDP period for the city public hospitals was defined as the period from October 2015 to September 2016, while the post-UZMDP period was defined as the period from October 2016 to September 2017. Difference-in-Difference negative binomial and Tobit regression models were employed to evaluate the impacts of the UZMDP on healthcare utilization and expenditures, respectively.

**Results:**

Four key findings flow from our assessment of the impacts of the UZMDP: first, outpatient and inpatient visits increased by 8.89 % and 9.39 %, respectively; second, average annual inpatient and outpatient drug expenditures fell by 4,349.00 CNY and 1,262.00 CNY, respectively; third, average annual expenditures on other categories of healthcare expenditures increased by 2,500.83 CNY, 417.10 CNY, 122.98 CNY, and 143.50 CNY for aggregate inpatient, inpatient diagnosis, inpatient laboratory, and outpatient medical consumables expenditures, respectively; and fourth, men and older individuals tended to have more inpatient and outpatient visits than their counterparts.

**Conclusions:**

Although the UZMDP was effective in reducing both inpatient and outpatient drug expenditures, it led to a sharp rise in other expenditure categories. Policy decision makers are advised to undertake efforts to contain the growth in total healthcare expenditures, in general, as well as to evaluate the offsetting effects of the policy on non-drug components of care.

## Background

During China’s planned economy era from 1949 to 1992 when free healthcare services were provided to every Chinese citizen, the Chinese government set drug prices below their costs and public healthcare facilities relied on government subsidies [1]. Because the Chinese government was unable to bear such a heavy financial burden from these subsidies, since 1950 it permitted public healthcare facilities to include a 15 % markup over the drug acquisition cost in the prices charged to customers [2–4]. Although this markup policy stabilized the finances of public healthcare facilities [2], it offered incentives to healthcare providers to over-prescribe drugs [5–8] and resulted in a rapid escalation of healthcare expenditures [9]. This scenario was summarized in a popular lament in China as “kanbingnan, kanbinggui,” or “insurmountable access barriers to health care, insurmountable high health costs” [10].

To restrain the rapid growth of healthcare expenditures, China unveiled an ambitious set of health reforms in 2009 [11], with a key component of these reforms being the zero-markup drug policy [8]. The aim of this policy was to discourage healthcare providers from over-prescribing drugs [3, 8, 12–14] by reducing drug prices and thus improving the availability and affordability of healthcare services [2, 15]. For the sake of smooth and stable implementation, the Chinese governments have adopted a step-by-step strategy to push forward the zero-markup drug policy. In 2009, primary healthcare institutions were required to remove the markups on drugs included in the Chinese National Essential Medicine List. Subsequently, the zero-markup policy was expanded to include county public hospitals in 2015, and in 2016, the zero-markup policy was applied to all drugs and also to city public hospitals [16].

Extensive research has investigated the effects of the zero-markup drug policy on primary healthcare institutions [14, 15, 17–20] and county public hospitals [21, 22], whereas there has been a paucity of studies examining the impacts of the zero-markup drug policy on city hospitals (also known as the universal zero-markup drug policy (UZMDP)) [16]. The adoption of the UZMDP has been shown to enhance healthcare utilization [23] and reduce drug expenditures [2, 8, 16, 23–28]. However, there are some studies that reported opposite findings by demonstrating a reduction in healthcare utilization [24, 26] and a rise in diagnosis, laboratory, medical consumables, or aggregate healthcare expenditures [2, 16, 25–28]. Additionally, much of the research to date has been carried out from the hospital perspective in order to evaluate the impacts of the UZMDP on hospital revenues per visit, per month, or per patient [2, 8, 16, 23–26, 28], whereas studies from a patient’s perspective were rare. As such, there is an opportunity to add to the literature by evaluating the comprehensive impacts of the UZMDP adoption on healthcare utilization and expenditures from a patient’s perspective. Specifically, the purpose of this study was to evaluate the impacts of the UZMDP on healthcare utilization as well as on aggregate and various components of healthcare expenditures.

### Conceptual framework

Our research was guided by Anderson’s health behavior model [29] that focuses on the determinants of healthcare utilization (and hence, healthcare expenditures) [30]. According to Anderson model, health policies are captured as a contextual enabling factor thereby impacting health behaviors, especially the use of healthcare services [29]. In the present context, the UZMDP has been shown to be associated with inpatient and outpatient service utilization, although the direction of such an association is debated [23, 24, 26]. Some studies have demonstrated that the UZMDP resulted in a large reduction in inpatient and outpatient drug expenditures [2, 8, 16, 23–28], though studies have also shown that the UZMDP offered incentives to healthcare providers to seek new, potentially inappropriate, forms of revenue to recoup lost drug revenues [2, 16, 25–28]. It is therefore expected that the UZMDP will not only have direct impacts on drug expenditures but have spillover effects on other expenditure categories, such as diagnosis, laboratory, medical consumables, and aggregate expenditures for outpatient and inpatient services. Based on these considerations, our study hypothesized that the implementation of the UZMDP would have impacts on patients’ inpatient and outpatient service utilization as well as on both aggregate healthcare expenditures and its various components (including drug, diagnosis, laboratory, and medical consumables expenditures).

## Methods

### Setting and data sources

Changde city is located in central China and has a population of over 1.5 million in 2019 [31]. Staring from October 2016, 13 city public hospitals in Changde city were designated by the Changde municipal government as pilot institutions to implement the UZMDP [32]. These hospitals are the main providers of healthcare services in Changde city as they accounted for almost 60 % of the total number of beds and medical staff in the city [33]. Data were extracted from the Changde Municipal Human Resource and Social Security Bureau that links a wide range of databases to patient-level data. The databases used in our research include healthcare expenditures and demographic data on patients in Changde city who were covered by public health insurance programs from October 2015 to September 2017.

### Study design and participants

Experimental research design is often recommended as the gold standard to understand the impacts of health policies [34]. Since it is not feasible to randomize policy intervention to individual patients, a pre-post design that attempts to establish a cause-and-effect relationship without random assignment was employed [15]. In line with previous research [15], patients who visited the pilot hospitals at least once during the pre-UZMDP period (from October 2015 to September 2016) and at least once during the post-UZMDP period (from October 2016 to September 2017) and did not visit the non-UZMDP institutions were assigned to the intervention group, whereas the comparison group comprised those who visited the non-UZMDP institutions at least once during the pre-UZMDP period and at least once during the post-UZMDP period and did not visit the pilot hospitals. We constructed an inpatient and an outpatient sample comprising 13,623 and 24,141 patients over the course of the two periods, respectively.

### Measures of variables

Inpatient and outpatient services utilization and expenditures were the outcomes of interest. Since this research was carried out from a patient’s perspective, the unit of analysis would be individual patients. Inpatient and outpatient service utilization were measured by the yearly count of inpatient and outpatient visits [15]. Healthcare expenditures were measured by annual aggregate, drug, diagnosis, laboratory, and medical consumables expenditures for outpatient and inpatient services [27]. We included the implementation of the UZMDP as a dummy independent variable [2, 8, 16, 23–28]. Additionally, age (year) [27, 35–37], annual income (0 - 20,000 CNY, 20,000 - 40,000 CNY, 40,000 - 60,000 CNY, or ≥ 60,000 CNY) [27, 35, 37], gender (female or male) [27, 36], and type of health insurance (health insurance for residents, health insurance for retired veteran cadres, health insurance for disabled soldiers, or health insurance for employees) [27, 35, 36] were included in our study as covariates.

### Statistical analyses

The Difference-in-Difference (DID) regression technique has been widely applied by previous studies to present detailed analyses of the impacts of the UZMDP on inpatient and outpatient visits and healthcare expenditures [16, 22]. Since the yearly counts of inpatient and outpatient visits were constructed as our dependent variables, the classic Poisson regression model was a candidate for modelling these count data [38]. However, the classical Poisson regression model is often of limited use because empirical count variables typically exhibit over-dispersion, i.e., the variances of the count variables exceed their means [39]. Under these conditions, a negative binomial model which has the same mean structure as the classical Poisson model but allows for dispersion was selected to evaluate the impacts of the UZMDP on inpatient and outpatient visits [39]. For values of healthcare expenditures that were left censored at 0, conventional regression models would produce biased estimates, so Tobit regression techniques that are designed to model censored data were selected [15].

To check for the validity of model application, a series of regression diagnostic tests were adopted. A dispersion test was performed to check whether our count dependent variables were over-dispersed. The likelihood ratio test was conducted to examine the fitness of the negative binomial model in comparison with the classical Poisson model. We performed a chi-squared test and used McFadden’s pseudo R^2^ to explore the goodness-of-fit of the healthcare utilization and expenditures models, respectively.

We also conducted a sensitivity analysis to check the robustness of model outputs. Due to limited access to data, only four covariates (including age, gender, annual income, and type of health insurance) were included in the DID model. To control the impacts of time-invariant unobserved omitted variables, we further constructed the fixed effects (FE) models where first differenced variables were constructed. This allows us to compare the findings of the DID model with the FE model. All analyses of our research were conducted using R 3.6.3 statistical software [40].

## Results

### Descriptive results

Table 1 reports the characteristics of the study sample. The mean number of annual inpatient admissions for the sampled inpatients was 1.98 with a standard deviation (SD) of 1.70. Inpatient annual aggregate, drug, diagnosis, laboratory, and medical consumables expenditures averaged at 16,280.57 CNY, 6,161.17 CNY, 1,573.40 CNY, 1,005.83 CNY, and 854.30 CNY, respectively. Most of our sampled inpatients were male (53.05 %), employed (71.12 %), and had annual income between 20,000 and 40,000 CNY (67.28 %). Meanwhile, the mean number of annual outpatient visits for the sampled outpatients was 3.75 (SD = 4.09). Outpatient annual aggregate, drug, diagnosis, laboratory, medical consumables expenditures averaged at 1,325.59 CNY, 458.04 CNY, 52.18 CNY, 20.92 CNY, and 12.81 CNY, respectively. Unlike the sampled inpatients, most of the outpatients in our study were female (57.35 %).

**Table 1:** Descriptive statistics for the sample patients

		*Inpatient sample (n = 27,246)*				*Outpatient sample (n = 48,282)*			
		*Mean*	*SD*	*Min*	*Max*	*Mean*	*SD*	*Min*	*Max*
***Dependent variables***	**Number of annual visits**	1.982	1.696	1	26.000	3.754	4.089	1	75.000
	**Annual aggregate expenditures**	16,280.572	28,727.542	10	1,191,614.900	1,325.591	4,983.619	0	152,774.210
	**Annual drug expenditures**	6,161.174	13,505.055	0	380,865.680	458.037	2,111.263	0	82,278.790
	**Annual diagnosis expenditures**	1,573.404	4,528.603	0	316,404.000	52.183	145.871	0	3,238.000
	**Annual laboratory expenditures**	1,005.827	1,365.669	0	24,207.000	20.917	87.693	0	2,837.000
	**Annual medical consumables expenditures**	854.300	3,258.344	0	145,023.910	12.812	120.337	0	8,938.000
***Independent variable***	**Implementation of UZMDP** Yes	19,220 (70.542% )				36,634 (75.875%)			
	**Implementation of UZMDP** No	8,026 (29.458%)				11,648 (24.125%)			
***Covariates***	**Age** (year)	61.392	19.592	0	117	51.568	16.399	1	117
	**Annual income** 0 - 20,000 CNY	3,622 (13.294%)				3,530 (7.311%)			
	**Annual income** 20,000 - 40,000 CNY	18,331 (67.280%)				24,168 (50.056%)			
	**Annual income** 40,000 - 60,000 CNY	3,591 (13.180%)				10,844 (22.460%)			
	**Annual income** ≥ 60,000 CNY	1,702 (6.247%)				9,740 (20.173%)			
	**Gender** Female	12,792 (46.950%)				27,688 (57.346%)			
	**Gender** Male	14,454 (53.050%)				20,594 (42.654%)			
	**Type of health insurance** Residents^a^	7,575 (27.802%)				1,853 (3.838%)			
	**Type of health insurance** Retired veteran cadres^b^	257 (0.943%)				740 (1.533%)			
	**Type of health insurance** Disabled soldiers	36 (0.132%)				0 (0.000%)			
	**Type of health insurance** Employees^a^	19,378 (71.122%)				45,689 (94.629%)			

^a^Residents and employees were covered by the Urban and Rural Resident Basic Medical Insurance Scheme and the Urban Medical Employee Medical Insurance respectively

^b^Veteran cadres are CCP (China’s Community Party) members who participated revolutionary wars before 1949

Table 2 describes the descriptive characteristics of the sample by the UZMDP adoption. Among the inpatient sample, 8,026 inpatients (29.46 %) were in the comparison group, while the remaining 19,220 inpatients (70.54 %) constituted the intervention group. Among the outpatient sample, about a quarter of outpatients (*N* = 11,648; *P* = 24.12 %) were assigned to the comparison group, while the rest of the patients (*N* = 36,634; *P* = 75.88 %) constituted the intervention group. The patients in the comparison group were found to have more inpatient and outpatient visits than their counterparts in the intervention group (*p*-value < 0.01). Likewise, the patients in the comparison group were found to have higher annual aggregate and drug expenditures than those in the intervention group (*p*-value < 0.01). In contrast, the patients in the comparison group tended to have lower annual diagnosis and laboratory expenditures than those in the intervention group (*p*-value < 0.01).

**Table 2:** Descriptive statistics for the sample patients by the UZMDP adoption

	*Inpatient sample (n = 27,246)*			*Outpatient sample (n = 48,282)*		
	*Comparison group*	*Treatment group*	*P-value*	*Comparison group*	*Treatment group*	*P-value*
**Number of patients**	8,026 (29.46)	19,220 (70.54)		11,648 (24.12)	36,634 (75.88)	
**Number of annual visits**	2.50 (2.15)	1.76 (1.41)	< 0.001	4.06 (5.13)	3.66 (3.69)	< 0.001
**Annual aggregate expenditures**	18,677.56 (35,528.89)	15,279.62 (25,286.93)	< 0.001	2,318.99 (7741.01)	1,009.73 (3,642.55)	< 0.001
**Annual drug expenditures**	8,613.33 (20,868.09)	5,137.19 (8,553.18)	< 0.001	932.81 (4176.39)	307.08 (484.50)	< 0.001
**Annual diagnosis expenditures**	524.39 (3,160.27)	2,011.46 (4,924.52)	< 0.001	7.86 (48.51)	66.28 (162.71)	< 0.001
**Annual laboratory expenditures**	401.34 (892.66)	1,258.25 (1,447.37)	< 0.001	4.25 (40.94)	26.22 (97.40)	< 0.001
**Annual medical consumables expenditures**	537.15 (3,602.22)	986.74 (3,093.98)	< 0.001	14.60 (175.57)	12.24 (96.35)	0.065
**Age** (year)	61.49 (16.00)	61.35 (20.91)	0.590	55.71 (16.84)	50.25 (16.04)	< 0.001
**Gender** Female	4,012 (0.50)	8,780 (0.46)	< 0.001	5,228 (0.45)	22,460 (0.61)	< 0.001
**Gender** Male	4,014 (0.50)	10,440 (0.54)		6,420 (0.55)	14,174 (0.39)	
**Annual income** 0 - 20,000 CNY	1,136 (0.14)	2,486 (0.13)	< 0.001	1,592 (0.14)	1,938 ( 0.05)	< 0.001
**Annual income** 20,000 - 40,000 CNY	5,182 (0.65)	13,149 (0.68)		7,126 (0.61)	17,042 (0.47)	
**Annual income** 40,000 - 60,000 CNY	1,186 (0.15)	2,405 (0.13)		1,366 (0.12)	9,478 (0.26)	
**Annual income** ≥ 60,000 CNY	522 (0.07)	1,180 (0.06)		1,564 (0.13)	8,176 (0.22)	
**Type of health insurance** Residents	1,836 (0.23)	5,739 (0.30)	< 0.001	50 (0.004)	1,803 (0.05)	< 0.001
**Type of health insurance** Retired veteran cadres	71 (0.01)	186 (0.01)		740 (0.06)	-	
**Type of health insurance** Disabled soldiers	7 (0.001)	29 (0.002)		-	-	
**Type of health insurance** Employees	6,112 (0.76)	13,266 (0.69)		10,858 (0.93)	34,831 (0.95)	

For continuous variables: mean (SD), while for categorical variables: mean (%)

Table 3 reports the descriptive characteristics of the sample by period. In the pre-UZMDP period, inpatient annual aggregate, drug, diagnosis, laboratory, and medical consumables expenditures averaged at 16881.17 CNY, 4442.70 CNY, 1447.27 CNY, 968.76 CNY, and 818.19 CNY, respectively. Meanwhile, outpatient annual aggregate, drug, diagnosis, laboratory, and medical consumables expenditures averaged at 1274.00CNY, 213.24 CNY, 47.63 CNY, 21.66 CNY, and 9.81 CNY, respectively. The number of annual inpatient admissions and inpatient annual aggregate expenditure in the post-UZMDP period were smaller than those in the pre-UZMDP period (*p*-value < 0.01).

**Table 3:** Descriptive statistics for the sample patients by period

	*Inpatient sample (n = 27,246)*			*Outpatient sample (n = 48,282)*		
Mean (SD)	Pre-UZMDP period	Post-UZMDP period	*P*-value	Pre-UZMDP period	Post-UZMDP period	*P*-value
**Number of annual visits**	2.05 (1.79)	1.91 (1.59)	<0.001	3.69 (4.15)	3.82 (4.02)	<0.001
**Annual aggregate expenditures**	16,881.17 (29,010.76)	15,679.98 (28,429.88)	0.001	1,274.00 (4,762.41)	1,377.18 (5,195.01)	0.023
**Annual drug expenditures**	4,442.70 (7,808.90)	7,879.65 (17,259.80)	<0.001	213.24 (396.81)	702.83 (2,939.00)	<0.001
**Annual diagnosis expenditures**	1,447.27 (4,017.63)	1,699.53 (4,984.45)	<0.001	47.63 (137.76)	56.73 (153.43)	<0.001
**Annual laboratory expenditures**	968.76 (1,320.86)	1,042.90 (1,408.13)	<0.001	21.66 (91.28)	20.18 (83.95)	0.064
**Annual medical consumables expenditures**	818.19 (3,172.11)	890.41 (3,342.08)	0.067	9.81 (110.17)	15.81 (129.65)	<0.001

### Regression results

Table 4 reports the impacts of the UZMDP on inpatient visits and expenditures. The estimated coefficient of the UZMDP on inpatient visits was 0.09 with a 95 % CI between 0.05 and 0.13. The estimated coefficient of the UZMDP on inpatient drug expenditures was -4349.00 with a 95 % CI between -5070.84 and -3628.10. In contrast, the UZMDP was found to boost aggregate annual inpatient expenditures (coefficient: 2500.83; 95 % CI: 1019.48 and 3982.17), diagnosis expenditures (coefficient: 417.10; 95 % CI: 135.01 and 699.21), and laboratory expenditures (coefficient: 122.98; 95 % CI: 41.41 and 204.56). The positive association between the adoption of the UZMDP and inpatient medical consumable expenditures was also found, although such association was not statistically significant.

**Table 4:** The impacts of UZMDP on inpatient services utilization and expenditures (DID model)

	*Inpatient admissions*		*Aggregate*		*Drug*		*Diagnosis*		*Laboratory*		*Medical consumables*	
	***Estimate***^a^	***Se***	***Estimate***	***Se***	***Estimate***	***Se***	***Estimate***	***Se***	***Estimate***	***Se***	***Estimate***	***Se***
**Intercept**	0.772***	0.025	18,479.543***	912.982	3,029.000***	444.800	-4,057.000***	174.200	-748.076***	49.682	-1,762.000***	124.500
**UZMDP adoption**	0.089***	0.020	2,500.827***	755.767	-4,349.000***	368.100	417.100**	143.900	122.984**	41.621	117.300	104.700
**Year**	-0.126***	0.016	-3,040.138***	634.838	7,141.000***	310.200	131.500	124.000	24.121	35.803	149.700	90.370
**Group**	-0.390***	0.014	-4,523.301***	535.535	-501.500	262.700	3,056.000***	102.900	1,294.083***	29.719	1,768.000***	75.070
**Age** (year)	0.002***	0.000	69.153***	9.696	34.750***	4.698	25.900***	1.814	7.027***	0.514	4.716***	1.281
**Annual income**												
0 - 20,000 CNY	REF	REF	REF	REF	REF	REF	REF	REF	REF	REF	REF	REF
20,000 - 40,000 CNY	-0.006	0.015	-6,258.998***	551.338	-2,835.000***	266.800	-7.744	100.300	-89.806**	29.188	16.440	73.150
40,000 - 60,000 CNY	-0.010	0.018	-3,079.387***	683.493	-946.800**	331.000	-526.300***	125.100	-259.216***	36.431	-211.400*	91.170
≥ 60,000 CNY	-0.084***	0.024	-3,511.692***	866.390	-2,185.000***	421.500	-157.800	158.900	-349.094***	46.375	-132.500	115.700
**Gender**												
Female	REF	REF	REF	REF	REF	REF	REF	REF	REF	REF	REF	REF
Male	0.066***	0.010	2,592.978***	351.871	851.200***	170.600	218.900***	64.320	86.178***	18.654	39.780	46.610
**Type of health insurance**												
Residents	REF	REF	REF	REF	REF	REF	REF	REF	REF	REF	REF	REF
Employees	0.078***	0.012	1,037.861*	451.458	655.200**	218.900	1,130.000***	82.820	313.736***	24.042	575.800***	60.230
Retired veteran cadres	0.284***	0.047	12,443.604	1,899.796	4,975.000***	915.400	2,916.000***	342.200	699.456***	99.731	579.300*	254.000
Disabled soldiers	0.455	0.111	-7,875.390	4,778.139	-2,587.000	2,307.000	873.300	846.800	285.421	247.638	-795.800	644.600
Regression diagnostics^b^	0.000/1.000/0.000		0.021		0.002		0.004		0.012		0.003	

^a^Significance codes: *** ≤ 0.001, ** ≤ 0.01, * ≤ 0.05

^b^Regression diagnostics: the p-values of the Over-dispersion test, the Chi-squared test, and the Likelihood ratio test were reported for the healthcare utilization models, while the McFadden’s pseudo R^2^ was reported for the healthcare expenditures models

Table 5 reports the impacts of the UZMDP on outpatient visits and expenditures. The UZMDP was found to increase outpatient visits while it reduced associated drug expenditures. When the UZMDP was implemented, the number of annual outpatient visits increased by 9.39 % (95 % CI: 0.06 and 0.13) whereas annual outpatient drug expenditures fell by -1262.00 CNY (95 % CI: -1345.86 and -1177.15). The UZMDP was shown to raise annual expenditures for outpatient medical consumables (coefficient: 143.50; 95 % CI: 112.00 and 174.92). The UZDMP was also found to reduce outpatient laboratory expenditures but raise outpatient total and diagnosis expenditures, although these associations were not statistically significant in our study.

**Table 5:** The impacts of UZMDP on outpatient services utilization and expenditures (DID model)

	*Outpatient visits*		*Total*		*Drug*		*Diagnosis*		*Laboratory*		*Medical consumables*	
	***Estimate***^a^	***Se***	***Estimate***	***Se***	***Estimate***	***Se***	***Estimate***	***Se***	***Estimate***	***Se***	***Estimate***	***Se***
**Intercept**	-0.073	0.037	1,478.000***	169.1	-1,254.000***	80.74	-1,119.000***	34.22	-627.924***	21.896	-1,766	1,322
**UZMDP adoption**	0.094***	0.018	158.3	93.62	-1,262.000***	43.04	29.2	15.1	-12.033	11.745	143.500***	16.05
**Year**	-0.035*	0.015	-23.01	81.56	1,568.000***	37.48	13.54	14.15	17.507	11.049	-33.500*	15.3
**Group**	0.041**	0.013	-81.460***	68.05	630.700***	31.72	343.000***	11.07	222.769***	8.685	217.2	11.34
**Age** (year)	0.001***	0.000	8.763***	1.366	1.802**	0.631	-0.926***	0.175	-1.691***	0.132	-0.345*	0.163
**Annual income**												
0 - 20,000 CNY	REF	REF	REF	REF	REF	REF	REF	REF	REF	REF	REF	REF
20,000 - 40,000 CNY	-0.160***	0.016	-671.100***	87.76	-200.800***	40.23	-3.361	10.98	-4.903	8.489	-39.150***	9.828
40,000 - 60,000 CNY	-0.012	0.018	-848.600***	96.99	-41.35	44.39	-3.832	11.99	-3.874	9.243	-19.86	10.73
≥ 60,000 CNY	0.080***	0.018	-585.600***	98.39	119.200**	45.01	-1.436	12.16	-9.578	9.359	11.31	10.83
**Gender**												
Female	REF	REF	REF	REF	REF	REF	REF	REF	REF	REF	REF	REF
Male	0.037***	0.008	456.500***	42.14	26.41	19.4	-49.670***	5.284	-36.485***	4.039	-20.150***	4.827
**Type of health insurance**												
Residents	REF	REF	REF	REF	REF	REF	REF	REF	REF	REF	REF	REF
Employees	1.310***	0.029	-435.600***	111.8	412.400***	55.39	602.400***	28.65	276.711***	16.413	1,250	1,322
Retired veteran cadres	2.627***	0.042	17,400.000***	217.2	10,590.000***	99.71	-544.5	1,627	-509.056	1,718.69	199.1	4,709
Regression diagnostics^b^	0.000/1.000/0.000		0.013		0.026		0.021		0.019		0.023	

^a^Significance codes: *** ≤ 0.001, ** ≤ 0.01, * ≤ 0.05

^b^Regression diagnostics: the p-values of the Over-dispersion test, the Chi-squared test, and the Likelihood ratio test were reported for the healthcare utilization models, while the McFadden’s pseudo R^2^ was reported for the healthcare expenditures models

Age was found to be positively associated with inpatient visits and expenditures, while men tended to have more inpatient visits than their counterparts. Individuals covered by the Urban Medical Employee Medical Insurance scheme were found to have more inpatient visits than those covered by the Urban and Rural Resident Basic Medical Insurance Scheme. Meanwhile, men and older individuals tended to have more outpatient visits than their counterparts.

The regression diagnostics were reported in Tables 4 and 5. The p-value for the over-dispersion test was smaller than 0.05, implying the presence of over-dispersion in the count dependent variables. The likelihood ratio test demonstrated that the negative binomial model was more appropriate to use for estimation than the classical Poisson model (p-value < 0.05). The p-values of the chi-squared test for healthcare utilization models were not statistically significant, implying that these models fitted well. The outpatient drug expenditures model was found to have the best goodness-of-fit.

### Results of sensitivity analysis

Table 6 reports the results of the sensitivity analysis. The coefficients of the UZMDP on inpatient and outpatient visits in the FE model was larger than those in the DID model. The UZMDP led to a 24.76 % (95 % CI: 0.18 and 0.31) and 38.64 % (95 % CI: 0.27 and 0.50) growth in the annual number of inpatient and outpatient visits, respectively. The impacts of the UZMDP on healthcare expenditures exhibited similar trends in the FE model as those observed in the DID model. Specifically, the UZMDP was found to reduce annual inpatient (coefficient: -1,338.00; 95 % CI: -1,723.19 and -952.80) and outpatient (coefficient: -1,516.00; 95 % CI: -1,631.90 and -1,400.01) drug expenditures. The UZMDP was found to increase all the remaining expenditures. These results demonstrate that the findings of our core analysis were robust.

**Table 6:** The impacts of UZMDP on healthcare services utilization and expenditures (FE model)

	***Inpatient admissions***		***Total***		***Drug***		***Diagnosis***		***Laboratory***		***Medical consumables***	
	***Estimate***	***Se***	***Estimate***	***Se***	***Estimate***	***Se***	***Estimate***	***Se***	***Estimate***	***Se***	***Estimate***	***Se***
**Intercept**	-0.309***	0.027	6,801.300***	222.200	3,981.000***	166.000	-2,270.000***	95.980	-461.000***	28.750	-1,731.000***	73.410
**UZMDP adoption**	0.248***	0.032	669.800*	264.600	-1,338.000***	196.500	3,099.000***	108.900	1,299.000***	32.680	2,055.000***	82.980
**R**^**2**^	0.425 %		0.047 %		0.019 %		0.600 %		0.920 %		0.339 %	
	***Outpatient visits***		***Total***		***Drug***		***Diagnosis***		***Laboratory***		***Medical consumables***	
	***Estimate***	***Se***	***Estimate***	***Se***	***Estimate***	***Se***	***Estimate***	***Se***	***Estimate***	***Se***	***Estimate***	***Se***
**Intercept**	-0.161**	0.051	-13.210	32.050	430.400***	51.580	-648.817***	13.379	-442.521***	9.784	-639.507***	14.239
**UZMDP adoption**	0.386***	0.059	153.410***	36.790	-1,516.000***	59.160	348.593***	11.852	189.365***	8.212	372.503***	13.129
**R**^**2**^	0.177 %		0.072 %		0.219 %		1.343 %		1.166 %		1.761 %	

^a^Significance codes: *** ≤ 0.001, ** ≤ 0.01, * ≤ 0.05

## Discussion

We constructed negative binomial and Tobit regression models to evaluate the impacts of the UZMDP on healthcare utilization and expenditures. The UZMDP was effective in promoting inpatient and outpatient service utilization and reducing associated drug expenditures after controlling for a set of covariates. Despite these promising outcomes, aggregate inpatient, inpatient diagnosis, inpatient laboratory, and outpatient medical consumables expenditures dramatically increased with the adoption of the UZMDP.

### The impacts of the UZMDP on drug utilization and expenditures

Our research found that the UZDMP promoted inpatient and outpatient service utilization while reduced drug expenditures, which is consistent with previous research [2, 8, 16, 23–28]. Our findings contrast with studies that focused on the impacts of zero-markup drug policy in primary healthcare institutions [15] and county public hospitals [22]. The role of city public hospitals in China may account for these observations. In China, public hospitals, which may provide almost 90 % of all inpatient and outpatient services, play the most important role in health care delivery [41]. Compared with county public hospitals, the service volumes of city public hospitals are usually much larger, and the medical services provided are generally more advanced and comprehensive [16]. Hence, although China has a three-tier healthcare system in which hospitals and primary healthcare institutions are supposed to provide specialized and preventive care, respectively [35], patients preferred hospital-based services [42]. For these reasons, the UZMDP should have far-reaching impacts on overall healthcare utilization and expenditures than the zero-markup drug policy pursued among primary healthcare institutions and county public hospitals. Our findings were also consistent with evidence from other countries [43, 44]. For instance, Godman et al. (2009) found increased utilization and reduced expenditures of generics following a combination of reforms that include initiatives aiming at lowering the reimbursed price of generics in Austria [43]. Additionally, Yoo et al. (2015) demonstrated that a drug price control policy in Korea reduced the pharmaceutical expenditures for patients while increased the utilization of drugs [44].

### The impacts of the UZMDP on components of healthcare expenditures

In line with prior studies [2, 16, 25–28], our research confirmed that the UZDMP was positively associated with total inpatient expenditures, inpatient diagnosis, inpatient laboratory, and outpatient medical consumables expenditures. These findings were also consistent with research that focuses on the zero-markup drug policy implemented in county public hospitals in China [21]. As concluded by a review conducted by Liu et al. (2021), although most of previous studies showed a considerable decrease in drug spending after the implementation of the zero-markup drug policy, the total hospital income sustained [45]. Likewise, Hsu et al. (2014) conducted a study in Taiwan and found that although the drug reimbursement price reductions reduced total government expenditures for oral antidiabetic medications, the substantial reductions in utilization of targeted products were offset by increases in non-targeted products [46]. Our findings were also in accordance with international evidence [47–49]. As suggested by a previous study, although many developing countries have drug price policies, in many cases the evidence does not support their effectiveness in increasing the availability of healthcare services [47]. For instance, Suh et al. (2018) demonstrated that a price cut reform in South Korea contained costs by immediately reducing the cost of pharmaceuticals; however, the savings were expected to be offset by a prescription shift from pharmaceuticals with price reduction to pharmaceuticals without [48]. Shepherd (2017) also concluded that price controls meant to lower drug spending for some consumers may end up harming all consumers because price controls may create incentives for manufacturers to charge higher prices to non-covered patients to offset the required discounts under Medicaid and Medicare [49]. In sum, these findings emphasized that confronted with a limit on drug price to revenues, healthcare providers may face incentives to recoup income losses by raising revenues from other components of care.

### Individual characteristics impacting healthcare utilization and expenditures

Our study demonstrated that age was positively associated with both inpatient and outpatient visits, which corroborates previous research [36]. Nevertheless, our study demonstrated that men tended to have more inpatient and outpatient visits than women, which is not consistent with a prior study [36]. This discrepancy could be explained by the more extensive sample used in that study which comprised 73,110 observations from 228 communities within 9 provinces in China, whereas our study was carried out in one Chinese city. This indicates that there may exist gender differences in health status and associated healthcare utilization patterns between and among different regions in China. Our study also provided some preliminary evidence on the differential influences of age, gender, income, and type of health insurance on miscellaneous components of inpatient and outpatient expenditures.

### Implications for drug price reforms

Our research findings offered some policy implications to future drug price reforms in China: First, we showed how the UZMDP promoted healthcare utilization and reduced drug expenditures. As such, the general use of the UZMDP in all city hospitals across China may help expand health service use and contain drug expenditures. To improve the availability and affordability of healthcare services, policies are also suggested to be proposed to mitigate the offsetting impacts of the UZMDP on non-drug components of care. Specifically, policy decision makers are suggested to balance the benefits and losses of various stakeholders. Particularly, the incentive and performance-based assessment mechanisms should be formed simultaneously to demotivate medical professionals from offering unnecessary healthcare services.

## Strengths and limitations

Our research makes two important contributions to the literature. Unlike most studies that reported the positive effects of the zero-markup policy implemented in primary healthcare institutions [13, 14, 17–20] and county public hospitals [21, 22], our study revealed that although the UZMDP reduced drug expenditures as expected, it led to a sharp rise in other expenditure categories. Our findings are of critical significance as they inform policy decision makers about the overall health impacts of the zero-markup drug policy, depending on different implementation settings. Additionally, most of previous studies were carried out from the hospital perspective [2, 8, 16, 23–26, 28] and tended to ignored the whole payment of one course of treatment. In comparison, our research evaluated the impacts of the UZMDP on both aggregate healthcare expenditures and its various components from the patient perspective; hence, targeted policy interventions can be proposed to reduce the unintended negative impacts of implementing the UZMDP.

There are several limitations to note. First, it may be difficult to generalize our findings as the study was limited to one Chinese city. The UZMDP may yield varying impacts in different cities, depending on the manner in which the UZMDP is implemented. Notwithstanding this limitation, our research included more than 20,000 patients and therefore was representative of citizens in Changde city. Second, because our sample data were restricted to only individuals who had public health insurance coverage from October 2015 to September 2017, we were unable to investigate how the UZMDP might affect patients with other forms of insurance coverage. In 2015, 48.44 % of Chinese citizen had pubic health insurance and this figure increased to 84.6 % by the end of 2017 [50–52]. Consequently, our findings should be generally applicable to most citizens of Changde concerning the impacts of UZMDP on healthcare utilization and expenditures. We recommended studies to include individuals across different administrative regions and covered by private insurance schemes, which may help to increase the external validity of research results. Finally, due to limited access to data, we were unable to evaluate some important covariates, such as patients’ health status [14]; however, this concern would have been greater if we had not constructed the FE model to address the potential missing variable bias [53]. Future studies with a more detailed tracking of patients may generate more precise estimates.

## Conclusions

This study demonstrated that the introduction of the UZMDP was associated with a rise in inpatient and outpatient service utilization and a reduction in drug expenditures. At the same time, there were significant spillover effects that resulted in an increase in other healthcare expenditures that offset the decline in drug expenditures. This study suggests policy decision makers may direct efforts to control the growth in total healthcare expenditures, in general, as well as to evaluate the offsetting effects of UZMDP on non-drug components of care.

## Data Availability

The databases used in this study is not publicly available. The data that support the findings of this study are however available from the authors (Xinping Sha, 2,532,331,526@qq.com) upon reasonable request and with permission of the Changde Municipal Human Resource and Social Security Bureau.

## Abbreviations

UZMDP: Universal zero-markup drug policy
DID: Difference-in-Difference
FE: Fixed effects
SD: Standard deviation
CNY: Chinese Yuan
